# Partitioning and Exocytosis of Secretory Granules during Division of PC12 Cells

**DOI:** 10.1155/2012/805295

**Published:** 2012-06-06

**Authors:** Nickolay Vassilev Bukoreshtliev, Erlend Hodneland, Tilo Wolf Eichler, Patricia Eifart, Amin Rustom, Hans-Hermann Gerdes

**Affiliations:** ^1^Interdisciplinary Center for Neurosciences (IZN), Department of Neurobiology, University of Heidelberg, INF 364, 69120 Heidelberg, Germany; ^2^Biotechnology Engineering Program, Mannheim University of Applied Sciences, Paul-Wittsack-Straße 10, 68163 Mannheim, Germany; ^3^Department of Biomedicine, University of Bergen, Jonas Lies vei 91, 5009 Bergen, Norway

## Abstract

The biogenesis, maturation, and exocytosis of secretory granules in interphase cells have been well documented, whereas the distribution and exocytosis of these hormone-storing organelles during cell division have received little attention. By combining ultrastructural analyses and time-lapse microscopy, we here show that, in dividing PC12 cells, the prominent peripheral localization of secretory granules is retained during prophase but clearly reduced during prometaphase, ending up with only few peripherally localized secretory granules in metaphase cells. During anaphase and telophase, secretory granules exhibited a pronounced movement towards the cell midzone and, evidently, their tracks colocalized with spindle microtubules. During cytokinesis, secretory granules were excluded from the midbody and accumulated at the bases of the intercellular bridge. Furthermore, by measuring exocytosis at the single granule level, we showed, that during all stages of cell division, secretory granules were competent for regulated exocytosis. In conclusion, our data shed new light on the complex molecular machinery of secretory granule redistribution during cell division, which facilitates their release from the F-actin-rich cortex and active transport along spindle microtubules.

## 1. Introduction

Secretory granules (SGs) are the hormone and neuropeptide containing organelles of neuroendocrine cells that release their content upon depolarization-induced, Ca^2+^-dependent exocytosis. The biogenesis and stimulated secretion of these high-copy number organelles have been intensively studied in various interphase cell models [1–5]. In the case of neuroendocrine PC12 cells, real-time studies revealed that shortly after their biogenesis at the *trans*-Golgi network, SGs undergo a unidirectional, microtubule-dependent transport to the plasma membrane (PM) [3]. Studies on insulin-secreting MIN6 cells identified kinesin-1 as the candidate motor protein for this transport step [6]. SGs of PC12 and MIN6 cells were also found to undergo a myosin Va-dependent movement and restriction in the F-actin-rich cortex, where they complete their maturation [7]. In interphase cells, the majority of SGs in PC12 cells is immobilized underneath the PM and is referred to as morphologically docked [3, 8, 9]. Numerous studies on the exocytosis of docked SGs revealed two main pools of SGs according to their response to stimulation, namely, the readily releasable pool and the reserve pool [10]. For chromaffin cells, it was shown that SGs, which are not consumed by exocytosis, are eventually removed from the cell cortex and replaced by newer ones [5].

In contrast to the frequent studies on SGs in interphase cells, the fate of these organelles during cell division has received little attention. In general, two basic partitioning strategies for the organelle inheritance during cell division have been proposed [11, 12]. The stochastic mode of inheritance is observed when the respective organelle is present in a high copy number (e.g., peroxisomes [13]). In this case cytoplasmic division ensures an approximately equal partitioning among daughter cells. In contrast, the ordered inheritance mode postulates that organelles with low copy number need to be distributed among daughter cells by an active mechanism involving cytoskeletal elements. The paradigm for the latter is the segregation of chromosomes during mitosis.

To our knowledge, only one study [14] has addressed the fate of SGs during cell division. Based on electron and immunofluorescence microscopy of fixed cells, the authors provided evidence for a microtubule-dependent reorganization of adrenocorticotropin-containing SGs during division of At20 cells [14]. In addition, several studies investigated whether the regular function of SGs is impaired during cell division. This revealed that the vesicular transport route from the endoplasmic reticulum to the Golgi compartment as well as the constitutive secretory pathway from the *trans* Golgi network to the PM appeared to slow down during mitosis [15–17]. Furthermore, regulated secretion of histamine and serotonin in rat basophilic cells was reduced 10-fold in dividing cells [18, 19]. Although the underlying mechanism for the observed effects during mitosis remained elusive, one favored explanation for the cessation of secretory processes is the inhibition of vesicle fusion with target membranes in mitotic cells [15, 18].

In this study, we examined the inheritance and the functionality of SGs during cell division by applying state-of-the-art labeling and imaging techniques. In particular, the use of two GFP fusion proteins to selectively label SGs and microtubules in dividing PC12 cells enabled us to follow the dynamics of these markers and to correlate the movement of SGs and the mitotic spindle in great spatial and temporal detail. Furthermore, to address the functionality of SGs, we monitored the regulated secretion of a luminal marker of SGs at the single granule level and determined the competence of SGs for regulated exocytosis at different stages of mitosis.

## 2. Results

### 2.1. The Number of Peripherally Localized SGs Is Reduced in Metaphase Cells

**Figure 1:**
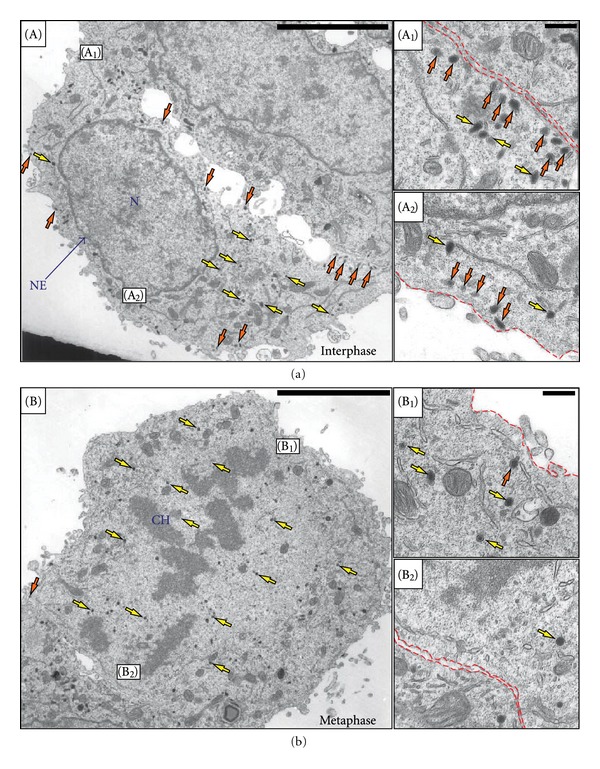
Ultrastructural analysis of the distribution of SGs in interphase and metaphase PC12 cells. (a) A typical interphase PC12 cell. The majority of SGs (size range of 80 to 150 nm) is peripherally localized (orange arrows). (b) A PC12 cell in metaphase. Almost all SGs are homogeneously distributed in the cytoplasm (yellow arrows), whereas peripheral SGs are rarely observed (orange arrows). Magnifications of the indicated regions in the main images (boxes in (A) and (B)) are shown on the right ((A_1_), (A_2_), (B_1_), and (B_2_)). The red dashed lines indicate the cell boundaries. N, nucleus; CH, chromosomes. Scale bars, main images 5 *μ*m, magnified images, 500 nm.

**Figure 2:**
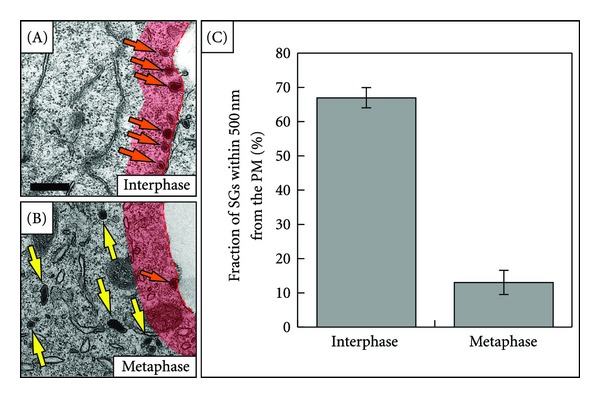
Statistical analysis of the amount of peripheral SGs in interphase and metaphase PC12 cells. The red translucent stripe in (A) and (B) images indicates a peripheral region adjacent to the PM, whose thickness is 500 nm. Arrows in (A) and (B) point to SGs that lie within the marked region (orange arrows) and SGs that do not belong to the marked region (yellow arrows). The graph in (C) summarizes a quantification of the fraction of SGs within the cortical 500 nm region of three interphase and three metaphase cells from different EM preparations. In interphase cells, 70 ± 3% of the total number of SGs (*n* = 227) were within the cortical region, compared to only 13 ± 4% of total SGs (*n* = 376) in metaphase cells (*P* = 0.0008, Student's *t*-test). Error bars in (C) represent standard deviations. Scale bar, 500 nm.

 Earlier studies have shown that the majority of SGs in interphase PC12 cells is localized in close proximity to the PM [3, 8, 9]. This was confirmed by an ultrastructural analysis of interphase PC12 cells (Figure 1), where SGs did not decorate the PM evenly but very often appear in discrete accumulations (Figures 1(A)–1(A_2_)). To analyze whether SGs retain their peripheral localization or undergo redistribution during cell division, we analyzed PC12 cell populations synchronized by a double thymidine block. At the ultrastructural level, mitotic cells can be distinguished from interphase cells based on the condensed state of chromatin (Figure 1(B), CH-label) and the absence of an intact nuclear envelope (Figure 1(B), compare with Figure 1(A)). Mitotic cells appeared to contain a similar number of dense-core organelles as compared to interphase cells, consistent with the view that SGs are retained during mitosis. In metaphase cells, SGs were largely absent from the cellular periphery (Figures 1(B), 1(B_1_)) and the few SGs in close proximity to the PM were not in accumulations as in interphase cells, but single (Figures 1(B), 1(B_1_)). No site of preferential SG accumulation was observed, rather, SGs were almost evenly distributed in the cytoplasm, except those areas occupied by the chromosomes (Figure 1(B), CH-label). A quantification of the fraction of peripheral SGs showed that in interphase cells on average 70 ± 3% (±SD) of the total number of SGs are peripherally localized, compared to only 13 ± 4% (±SD) in metaphase cells (Figure 2(C)). This quantification indicates that the number of morphologically docked SGs is significantly reduced in metaphase as compared to interphase PC12 cells.

### 2.2. SGs Are Liberated from the Periphery at Prometaphase

**Figure 3:**
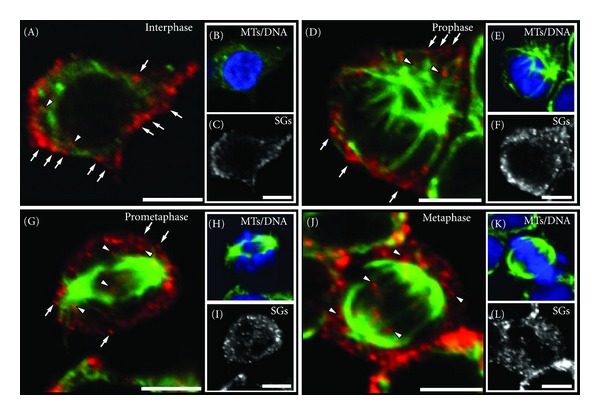
Distribution of SGs during early stages of PC12 cell division. Single confocal planes of representative immunostained PC12 cells at interphase ((A), (B), (C)), prophase ((D), (E), (F)), prometaphase ((G), (H), (I)), and metaphase ((J), (K), (L)) are shown as indicated. In interphase and prophase cells, SGs accumulate in the cell periphery ((A) and (D), arrows), while the number of nonperipherally localized SGs is significantly lower ((A) and (D), arrowheads,). During prometaphase, the number of peripheral SGs is reduced ((G), arrows), concurrent with an increase in the number of SGs in the cytoplasm ((G), arrowheads). SGs exhibited a nearly homogeneous distribution in cells at metaphase ((J), arrowheads) and no accumulation of SGs in the cell periphery could be detected. Scale bars, 5 *μ*m.

In order to distinguish between cells in prophase and prometaphase, we examined the distribution of SGs during early mitosis with immunofluorescence combined with confocal microscopy. Secretogranin II (SgII), a member of the granin family of regulated secretory proteins, which is efficiently sorted in SGs of PC12 cells, was used as an endogenous marker of SGs [20]. The pattern of distribution of SGs in interphase and prophase cells was very similar. The majority of SGs in these cells was peripherally localized in accumulations (Figures 3(A), 3(D), arrows) with only a small number of SGs found in the cytoplasm (Figures 3(A), 3(D), arrowheads). Interestingly, peripheral SGs in prometaphase cells were markedly reduced in frequency (Figure 3(G), arrows), paralleled by an increase of the number of SGs deeper inside the cell (Figure 3(G), arrowheads). SGs that remained in the periphery appeared as single puncta rather than as accumulations of punctuated signals, as observed in interphase and prophase (compare Figures 3(G) with 3(A) and 3(D)). In metaphase cells, the fraction of peripherally localized SGs was further reduced and the majority of SGs was almost homogeneously distributed across the cytoplasm, except those parts occupied by the chromosomes (Figures 3(J) and 3(L)). The latter is consistent with the ultrastructural analysis of metaphase PC12 cells.

**Figure 4:**
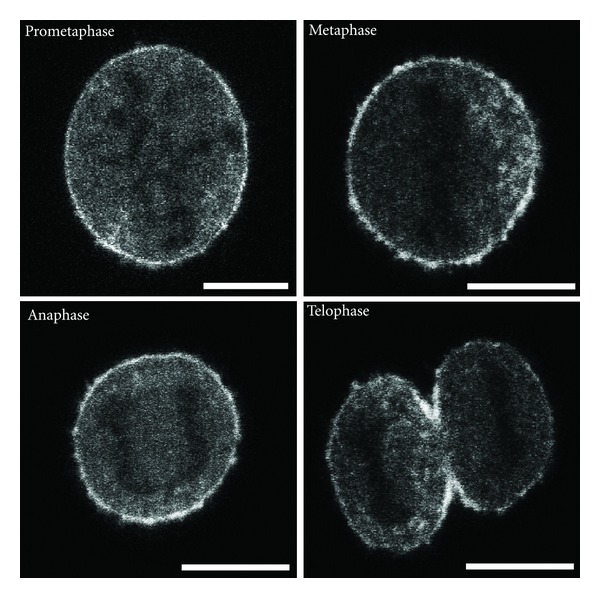
The F-actin cortex is maintained during division of PC12 cells. Actin-GFP was expressed in PC12 cells and dividing cells were imaged by confocal microscopy. Single focal planes through the middle of the cells are shown for different stages of cell division as indicated. Notably, through all phases of mitosis a pronounced peripheral, ribbon-like accumulation of actin-GFP was observed, indicating that the F-actin-rich cortex remained intact. Scale bar, 10 *μ*m.

To investigate if the progressive loss of peripheral SGs during prometa-/metaphase was due to depolymerization of the F-actin-rich cortex, dividing PC12 cells expressing *γ*-actin-EGFP fusion protein was analysed by time-lapse confocal microscopy. This showed a pronounced signal of cortical actin during all stages of cell division (Figure 4), which was comparable in strength to that known for interphase PC12 cells. Thus, the cortical F-actin appears to remain intact in dividing PC12 cells and the release of SGs from this area is likely to be caused by loss of their functional attachment to F-actin rather than depolymerization of F-actin itself.

### 2.3. SGs Accumulate in the Cell Midzone during Ana- and Telophase

**Figure 5:**
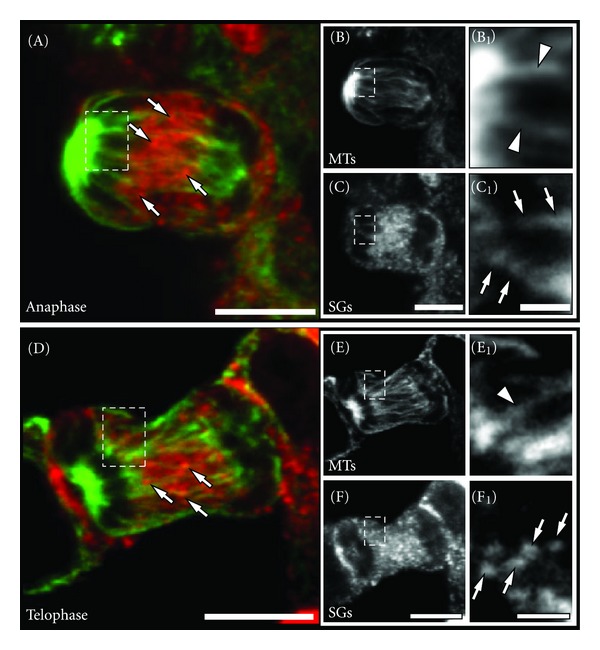
Distribution of SGs during late stages of mitosis. Single confocal planes of immunostained PC12 cells in anaphase ((A)-(C_1_)) and telophase ((D)-(F_1_)) are shown as indicated. Note that the majority of SGs during anaphase and telophase was found in the cell midzone ((A) and (D), arrows). A pronounced decoration of spindle microtubules ((E_1_) and (F_1_), green arrowheads) with SGs ((E_2_) and (F_2_), arrows) was frequently observed during these late stages of mitosis. Scale bars ((A), (C), (D), (F)), 5 *μ*m; ((C_1_), (F_1_)), 1 *μ*m.

**Figure 6:**
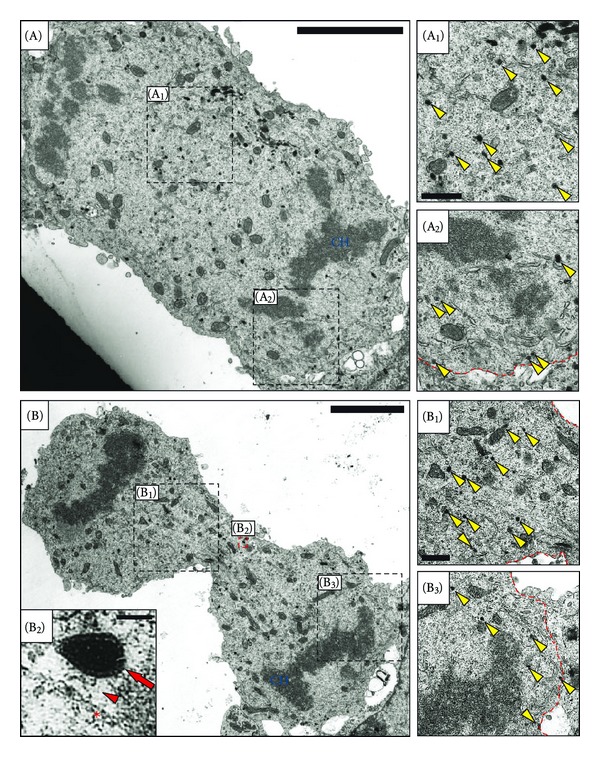
Ultrastructural analysis of the distribution of SGs during late stages of mitosis. Synchronized PC12 cell populations were processed for TEM. Shown are cells at anaphase (A) and telophase (B) and the respective magnified boxed areas on the right compare the density of SGs in the midzone ((A_1_)/(B_1_)) and the polar periphery ((A_2_)/(B_3_)). Please note that SGs are preferentially distributed in the cell midzone. Panel B_2_ is a magnification of the boxed region in panel B. Please note the close association of an SG (red arrow) with a microtubule fiber (red asterisk) and an apparent linker structure (red arrowhead) reminiscent of a motor protein. CH, chromosome. Scale bars, panels (A) and (B), 5 *μ*m; panels (A_1_), (A_2_), (B_1_), and (B_3_), 500 nm; panel (B_2_), 100 nm.

Similar immunofluorescence and confocal analysis of anaphase and telophase PC12 cells revealed that the majority of SGs was accumulated in the cell midzone (Figures 5(A)–5(C_1_) and Figures 5(D)–5(F_1_), resp.). As depicted in Figures 5(B_1_)/5(C_1_) and 5(E_1_)/5(F_1_), during these stages a frequent colocalisation of SGs (arrows) and spindle microtubules (arrowheads) was observed. This redistribution of SGs in the cell midzone was also evident at the ultrastructural level (please see Figure 6). SGs were also frequently observed in close proximity to microtubules at the ultrastructural level (Figure 6(B_2_)) with an apparent linker structure, reminiscent of a motor protein (Figure 6(B_2_), red arrowhead).

**Figure 7:**
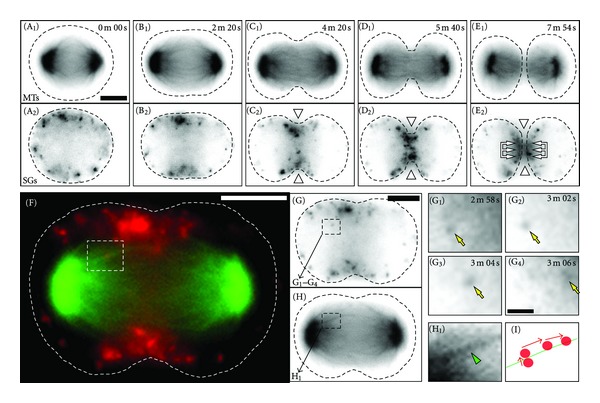
Dynamics of SGs during late stages of mitosis. PC12 cells were transfected with cDNAs coding for EGFP-tubulin (green) and hCgB-myc-DsRedExpress (red), synchronized and subjected to widefield fluorescence time-lapse microscopy. ((A_1_) to (E_2_)) An overview of the progression of a typical double-transfected PC12 cell through mitosis is shown. Images (A_1_)–(E_1_) and (A_2_)–(E_2_) represent the EGFP-tub and hCgB-myc-DsRedExpress channels, respectively. At the end of metaphase, SGs are homogeneously distributed in the cell ((A_1_)/(A_2_)). During anaphase, SGs temporarily accumulate at sites underneath the future cleavage furrow ((B_1_)/(B_2_)). During anaphase and telophase SGs gather in the cell midzone ((C_1_)/(C_2_) and (D_1_)/(D_2_)). At the end of telophase and beginning of cytokinesis SGs accumulate at the base of the intercellular bridge (grouped arrows in (E_2_)). Please note that the midbody in (E_1_)/(E_2_) is out of focus. ((F) to (I)) Tracks of moving SGs colocalise with microtubules. (F) A merged overview of a selected frame from the video sequence is shown. The single channel data for microtubules and SGs is shown in (G) and (H), respectively. Magnifications from the boxed regions in (F), (G), and (H) are shown to illustrate a punctuate structure positive for hCgB-myc-DsRedExpress that was tracked through four consecutive frames ((G_1_) to (G_4_), arrow) and whose trajectory overlapped with a spindle microtubule (H_1_). (I) A summary of the dynamic colocalisation of the tracked SG and the microtubule is shown. Elapsed time is given in minutes m and seconds s. Scale bars, (G_1_)–(I) 1 *μ*m, and all other images 5 *μ*m. The video sequence from which the presented images were taken is available as Supplementary Materials, Movie SM2.

The observed redistribution of SGs towards the cell midzone and the apparent colocalisation of SGs with spindle microtubules suggested that SGs might be actively transported into the cell midzone via microtubules. Therefore, we studied the dynamics of SGs relative to the microtubule cytoskeleton during mitosis by live-cell imaging. PC12 cells were cotransfected with cDNA constructs encoding hCgB-myc-DsRedExpress (a fluorescent marker of SGs) and EGFP-tubulin and were subjected to the established synchronization protocol and analyzed by wide-field fluorescence time-lapse microscopy. Mitotic cells expressing both exogenous fusion proteins were readily imaged as they progressed through all stages of mitosis (Figure 7 and Supplementary Materials available at doi:10.1155/2012/805295, Movie SM2, recording time 9 min 58 sec). At the end of metaphase, SGs were nearly homogeneously distributed in the cell cytoplasm except those parts occupied by the chromosomes (Figures 7(A_1_) and 7(A_2_)), which is in agreement with the data obtained from the confocal and ultrastructural analyses (Figure 1(B) and Figures 3(J), 3(L)). At the beginning of anaphase, SGs temporarily accumulated at a site underneath the future cleavage furrow (Figures 7(B_1_) and 7(B_2_)), whereas at the end of anaphase accumulations of SGs appeared also in the cell midzone (Figures 7(C_1_) and 7(C_2_)). At the end of telophase, the majority of SGs was found in the cell midzone (Figures 7(D_1_) and 7(D_2_)) and during cytokinesis, SGs accumulated at the base of the intercellular bridge (Figures 7(E_1_) and 7(E_2_)).

The putative interaction between SGs and spindle microtubules was also supported by these live-cell time-lapse imaging data. Tracks of SGs moving towards the cell midzone during anaphase and telophase frequently colocalized with spindle microtubules (Figures 7(F) to 7(I)). The average velocity of SGs with directed movements during anaphase was 0,23 ± 0,09 *μ*m/s (±SD). These observations are in agreement with the decoration of spindle microtubules with SGs which was documented by the confocal analysis of fixed anaphase and telophase PC12 cells (compare Figure 5).

### 2.4. SGs Accumulate at the Bases of the Intercellular Bridge and Are Absent from the Midbody

**Figure 8:**
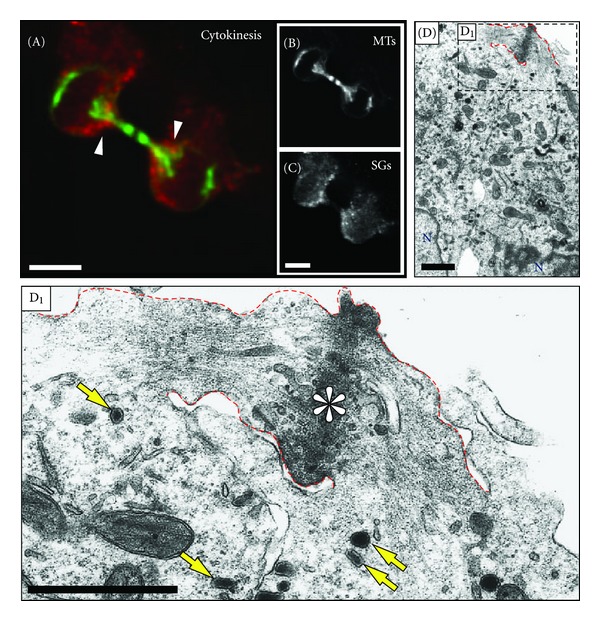
Distribution of SGs during cytokinesis. Single confocal images of immunostained PC12 cells in cytokinesis are shown as indicated. The main image (A) shows a merged presentation of microtubules and SGs; the single channels are shown in grayscale in (B) and (C), respectively. During cytokinesis, SGs were found to accumulate at the bases of the intercellular bridge ((A), arrowheads), but not in the midbody ((A) and (C)). ((D) and (D_1_)) PC12 cells in cytokinesis observed at the ultrastructural level. Shown in (D) are two PC12 daughter cells during cytokinesis which are connected by a midbody ((D), red dashed lines). The marked area (box in (D)) is magnified in (D_1_). Note that SGs are found only at the base of the intercellular bridge ((D_1_), yellow arrows). The asterisk in (D_1_) indicates the microtubule overlap region of the midbody. N, nucleus. Scale bars, (A)–(D), 5 *μ*m, (D_1_), 1 *μ*m.

Immunostained PC12 cells in cytokinesis were readily distinguished from other cells due to the tight bundle of microtubules present in the intercellular bridge and midbody. SGs in these cells accumulated at the bases of the intercellular bridge but were absent form the midbody (Figures 8(A)–8(C)). Consistent with these immunofluorescence data, SGs were frequently detected at the bases of the intercellular cytokinetic bridge and were absent from the “midbody matrix” region at the ultrastructural level (Figure 8(D_1_)). The latter observation was also confirmed by live-cell fluorescence imaging of dividing PC12 cells (Supplementary Materials, Movie SM1). Thus, the evidence gained with all three types of microscopical analyses indicated that SGs in PC12 cells accumulate at the bases of the intercellular bridge but are largely excluded from the midbody and the intercellular bridge itself. Time-lapse experiments of PC12 cells in cytokinesis also revealed movements of SGs towards and away from the bases of the intercellular bridge (Supplementary Materials, Movie SM3). The average velocity of these moving organelles was 0,34 ± 0,09 *μ*m/sec, which is consistent with a microtubule-dependent movement of SGs as shown previously for interphase PC12 cells [3].

### 2.5. SGs Undergo Regulated Exocytosis in Mitotic PC12 Cells

To address whether PC12 cells are competent for regulated exocytosis during cell division, the stimulated secretion of SgII was analyzed. Synchronized PC12 cells were subjected to a depolarization-induced stimulus by incubating them in high K^+^ growth medium, followed by a surface immunolabelling of SgII at 4°C (for details see Materials and Methods). As a control, nonstimulated cells (medium supplemented with 55 mM NaCl) were analyzed in parallel.

**Figure 9:**
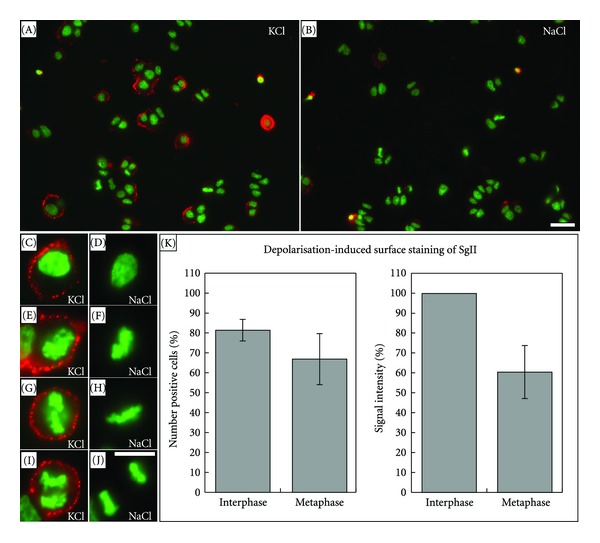
Analysis of regulated exocytosis in mitotic PC12 cells. Synchronized PC12 cell populations were incubated in growth medium supplemented with either 55 mM KCl ((A), (C), (E), (G), (I)) or 55 mM NaCl ((B), (D), (F), (H), (J)) and processed for immunofluorescence staining of surface-associated SgII (red) and DNA-staining (green). Interphase cells ((C) and (D)) and dividing cells at different stages of mitosis displayed prominent SgII surface staining under stimulating conditions ((E), prophase, (G), metaphase, (I), anaphase) but not under control conditions ((F), prophase, (H), metaphase, (J), anaphase). Scale bars: images (A) and (B), 10 *μ*m, (C) to (J), 5 *μ*m. (K) Quantitative analysis of the signal intensity of the SgII surface staining revealed that 81 ± 11% (±SD) of the interphase and 67 ± 26% (±SD) of the metaphase cells displayed a signal intensity above the background value determined under control conditions. In addition, the signal intensity of the SgII surface staining of metaphase cells was on average 61 ± 13% (±SD) of the mean intensity of interphase cells. For the evaluation, interphase and metaphase cells (*n* > 80, resp.) were randomly chosen from two independent experiments.

**Figure 10:**
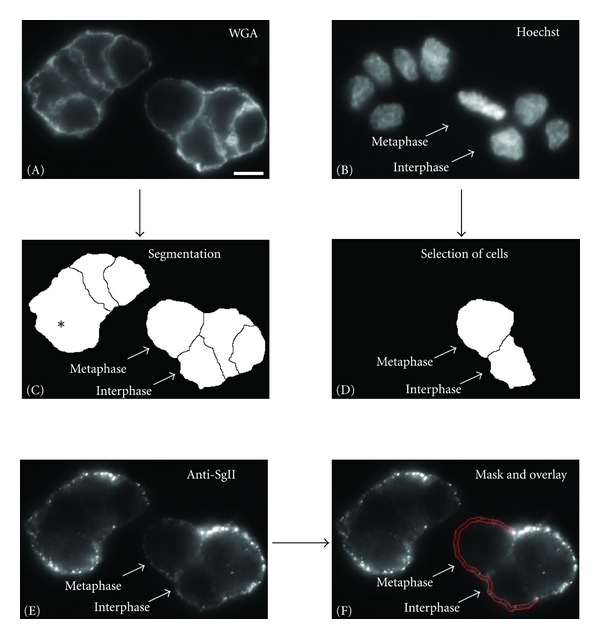
Quantification and comparison of the intensity of SgII surface staining of interphase and metaphase cells. PC12 cells were stained with WGA (A), Hoechst (B) and by immunocytochemistry for surface-associated SgII (E). Images displaying the WGA stain were used for segmentation (C). Asterisk in (C) indicates cell borders that are not detected. The Hoechst stain was used for the identification of metaphase and interphase cells (B). A mask was constructed for the quantification of the surface-associated SgII signal (red double-line in (F)) delineating the PM of PC12 cells located at the outside of the cell cluster. For more details please see Materials and Methods. Scale bar, 10 *μ*m.

Under control conditions, SgII was barely detectable at the surface of both interphase and mitotic PC12 cells (Figure 9(B)). However, stimulated cells exhibited a prominent surface staining for SgII (Figure 9(A)). Interestingly, also mitotic cells responded to the stimulus and frequently displayed newly exocytosed SgII at the cell surface (Figures 9(E), 9(G), 9(I)). It is of note that for both interphase and mitotic cells the surface signal intensity varied considerably from cell to cell (Figure 9(A)). For a quantitative and unbiased evaluation of the fraction of surface-stained cells as well as the intensity of the surface stain for both interphase and metaphase cells, we designed a semiautomatic algorithm for the calculation of the surface-staining signal intensity of user-selected cells (for details please see Figure 10 and Materials and Methods). This quantification revealed that approximately 80% of interphase and 65% of metaphase cells displayed signal intensities above background (Figure 9(K)). Furthermore, the average signal intensity of the surface-stain for metaphase cells was approximately 60% as compared to that of interphase cells (Figure 9(K)). Taken together, these quantitative data show that, although with an approximately 2-fold reduced efficiency, dividing cells are competent for regulated exocytosis.

## 3. Discussion

### 3.1. The Inheritance of SGs in PC12 Cells

**Figure 11:**
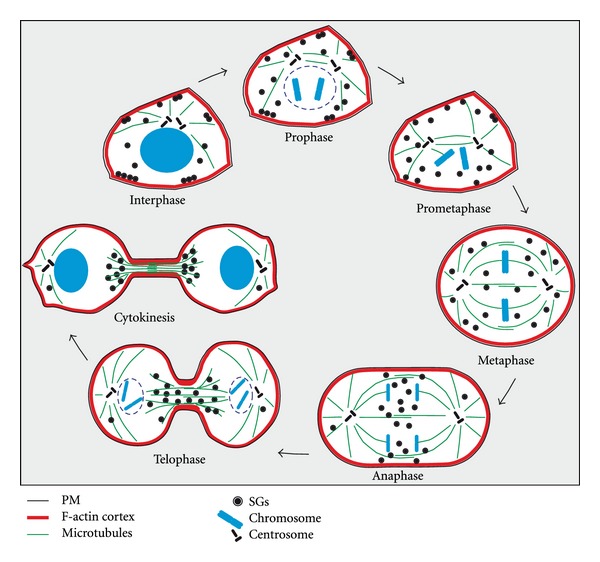
Model of SG partitioning during division of PC12 cells. The scheme summarizes the observed redistribution of SGs during cell division.

In this study, the partitioning and dynamics of SGs during cell division of neuroendocrine PC12 cells was comprehensively analyzed. The data presented here concur in substance with the drawn conclusions of Tooze and Burke on the inheritance of SGs in At20 cells [14] but, in extension of their study, provide detailed and direct insights into the dynamics and distribution of SGs during the division process. These straight insights were in particular gained from time-lapse imaging of SGs and microtubules during the consecutive phases of cell division. Based on our results, we propose the following model for the redistribution of SGs during cell division (Figure 11). During interphase, approximately 70% of all SGs are restricted to the F-actin-rich cell cortex. Accordingly, this fraction is referred to as morphologically docked (Figure 11). During prometaphase, the morphologically docked SGs are liberated from the cell cortex and spread throughout the cell. During metaphase, nearly all SGs are distributed nearly homogenously in the cytoplasm (Figure 11). At the onset of anaphase, SGs associate with and move along the spindle microtubules to reach the cell midzone. This results in an initial accumulation of SGs at the cell equator, juxtaposed to the nascent contractile ring. Subsequently, they spread from there over the entire cell midzone, occupying the space liberated by the pole-directed movement of the chromosomes (Figure 11). During cytokinesis, the progressive constriction of the contractile ring results in the exclusion of SGs from the midbody and their subsequent accumulation at the bases of the intercellular bridge. Although SGs exhibit bidirectional, presumably microtubule-dependent movement at the bases of the intercellular bridge, the accumulations at these sites are retained until late cytokinesis (Figure 11). The only significant difference in the inheritance modes of SGs in AtT20 and PC12 cells is observed during cytokinesis: SGs in AtT20 cells accumulate predominantly in the midbody, while SGs in PC12 cells accumulate at the bases of the cytokinesis bridge and are excluded from the midbody. This most probably reflects cell-specific differences in architecture and the biomechanics of the constriction of the midbody structure itself.

### 3.2. Redistribution of SGs during Cell Division

Two consecutive processes seem to orchestrate the redistribution of SGs during mitosis in PC12 cells: release of SGs from the cell periphery during prometaphase/metaphase and accumulation of SGs in the cell midzone during anaphase/telophase. In agreement with previous data showing that the retention of SGs in the cell cortex is dependent on the interaction between SGs and the F-actin cortex [3], the observed decrease of the cortical localization of SGs during mitosis implies that this interaction is abrogated. Our finding that the F-actin cortex of the PC12 cells is maintained during mitosis (please see Figure 4) suggests that the redistribution of SGs is not a consequence of the depolymerisation of the cortical F-actin. In a previous study, we reported that a dominant-negative mutant of myosin Va results in a strong abolishment of the peripheral restriction of SGs in interphase PC12 cells [7]. Hence, it is possible that upon entry into mitosis, the myosin Va-assisted capture of SGs in the F-actin cortex is abolished to result in the almost complete release of SGs during metaphase. This assumption is supported by a study of Rogers et al., which demonstrated a downregulation of myosin Va-dependent motility of melanosomes after treatment with metaphase-arrested *Xenopus* egg extracts [21].

The findings that SGs decorate and appear to move along spindle microtubules suggest that microtubule-dependent organelle transport occurs during mitosis. This contrasts the general dogma that microtubule-dependent motility of membrane-bound organelles is inhibited during mitosis [22]. One way to explain this discrepancy is that the experiments on cell-cycle-dependent regulation of organelle motility were carried out with interphase- and metaphase-arrested *Xenopus* extracts, whereas we observed an increased dynamic colocalisation of SGs and spindle microtubules during mitotic stages after metaphase (i.e., ana- and telophase). Thus, it is conceivable that microtubule-dependent organelle transport is blocked during metaphase and is reactivated during ana- and telophase, possibly in a cell- and/or organelle-specific manner.

### 3.3. Exocytosis of SGs during Cell Division

To date, secretory processes during cell division have been studied in several cell systems [15–18]. Apart from few exceptions, it seems generally accepted that fusion of vesicles with their target membrane is severely inhibited during mitosis. In this study, we reinvestigated this issue by focusing on SGs of the well-characterized PC12 cell system. By using a semiautomated algorithm to evaluate SG exocytosis in microscopy images (Figure 10), we found clear evidence for exocytosis of SGs in metaphase PC12 cells, which suggests that the fusion machinery of the regulated secretory pathway in mitotic PC12 cells is functional. The observed 2-fold reduction in regulated exocytosis as compared to interphase cells may be due to the observed loss in cortical restriction of SGs during metaphase leading to an unfavorable position for fusing with the PM.

Previous data on regulated exocytosis during mitosis indicated that stimulated release of histamine and serotonin in mitotic rat basophilic leukemia (RBL) cells is at least 10-fold reduced compared to interphase cells [18, 19]. Although both studies were carried out with the same cell line, they led to different conclusions as to why exocytosis was blocked. Oliver et al. attributed the secretion block to a failure in the transmembrane signaling during antigen-mediated stimulation and predicted that fusion of secretory vesicles with the PM of mitotic cells would be functional if the second messenger Ca^2+^ were within normal levels [19]. Hesketh et al. concluded that fusion of SGs with the PM in mitotic cells is impaired since they did not detect any significant difference in Ca^2+^ levels between interphase and mitotic cells [18]. Our data documenting only a 2-fold reduction in exocytosis of SGs in dividing versus interphase PC12 cells suggests that the degree of fusion of SGs with the PM is not severely impaired and hence secretory traffic might be functional during mitosis. This view is supported by studies on constitutive protein secretion, which indicated fusion competence of post-Golgi vesicles with the PM in mitotic Chinese Hamster Ovary (CHO) cells [16].

In conclusion, our study provides direct insights into the redistribution of SGs during cell division and sheds new light on the underlying complex molecular machinery promoting the release of SGs from the F-actin-rich cortex and their transport along spindle microtubules. Thus, despite the fact that SGs represent a high copy organelle, our findings support an ordered inheritance model involving active transport. Furthermore, our data suggest that during all stages of mitosis SGs are exocytosis-competent.

## 4. Materials and Methods

### 4.1. Culturing and Synchronization of PC12 Cell Populations

PC12 cells (rat pheochromocytoma cells, clone 251, [23]) were grown as described [24]. In order to increase the number of mitotic cells, one day after plating the cell populations were incubated in prewarmed growth medium supplemented with 4 mM thymidine (Sigma Aldrich Chemie GmbH, Munich, Germany) for 24 hours (block of cell division during S-phase). The cells were then washed with prewarmed growth medium once and subsequently incubated in growth medium (release of thymidine block). 18 hours thereafter we analyzed the cell populations by various types of microscopy. The latter synchronization protocol resulted in approximately 10% of the cells in mitosis.

### 4.2. Immunofluorescence

Indirect immunofluorescence of microtubules and SGs as well as staining of DNA was performed as described [3] except that the Moviol solution was supplemented with 1,5% 1,4-diazabicyclo[2.2.2]octane (DABCO) (Sigma Aldrich Chemie GmbH, Munich, Germany). The following primary antibodies were used: monoclonal antitubulin antibody (clone DM1A, Sigma Aldrich Chemie GmbH, Munich, Germany); polyclonal antibody 718 against rat secretogranin II as described [20]. Secondary antibodies were purchased from Jackson Immuno Research Lab, Inc., West Grove, PA, USA: donkey anti-mouse FITC (1 : 200) and goat anti-rabbit TRITC (1 : 500). DNA was stained with Hoechst dye 33258 (Molecular Probes, Inc., Eugene, OR, USA).

### 4.3. Cloning of pcDNA3-hCgB-myc-DsRedExpress and Transfection of PC12 Cells

To fluorescently label microtubules and SGs, PC12 cells were double-transfected with pEGFP-tubulin (Clontech Laboratories, Palo Alto, CA, USA) and pcDNA3/hCgB-myc-DsRedExpress. The latter cDNA construct was generated by amplifying the myc-DsRedExpress cDNA from pCMV-DsRedExpress (Clontech Laboratories, Palo Alto, CA, USA) with the (5′-3′) oligo sequences “ggc ggg ggt acc aga aca aaa act cat ctc aga aga gga tct gat ggc ctc ctc cga cg” and “ccc ccc gaa ttc cta cag gaa cag gtg g” as forward and reverse primers, respectively. The amplified DNA sequence was ligated into KpnI and EcoRI-digested pcDNA/hCgB-EGFP [3] by standard techniques. Actin-GFP DNA is described elsewhere [25]. PC12 cell populations were transfected by electroporation as previously described [1].

### 4.4. Fluorescence Microscopy

Wide-field time-lapse microscopy was performed on an Olympus IX70 equipped with a PCO CCD camera and Polychrome II light source (TillVision, Martinsried, Germany) and appropriate filter sets. Confocal fluorescence microscopy of fixed samples was performed on a Leica SP2 (Leica Microsystems, Mannheim, Germany). Confocal fluorescence time-lapse microscopy of actin-EGFP-transfected mitotic PC12 cells (Figure 4.) was carried out on a Leica SP5 (Leica Microsystems, Mannheim, Germany). Microscopy was performed with 100x/1.4 NA objectives. The spatial resolution of our imaging system was 200–250 nm, depending on the imaging mode and the wavelength used to excite the respective fluorophore.

### 4.5. Transmission Electron Microscopy

For TEM analysis, cells were prepared according to standard protocols. Briefly, synchronized PC12 cell populations were fixed in 2,5% glutaraldehyde, postfixed in 1% OsO_4_/1,5% K_4_Fe(CN_6_), dehydrated in a series of ethanol/propylene oxide treatments and mounted in “epoxy” resin. Ultrathin slices (80–100 nm) were prepared, contrasted with lead citrate and analyzed with an EM 10 CR Zeiss electron microscope at an acceleration voltage of 80 kV. For the statistical analysis (Figure 2), slices through the middle of three different cells in interphase and metaphase, obtained from three independent experiments, respectively, were evaluated.

### 4.6. Stimulation of PC12 Cells and Surface-SgII Staining

In order to analyze exocytosis of SGs during division of PC12 cells we used an established protocol as described by Pimplikar and Huttner [26]. Nontransfected cell populations were plated on PLL-coated (0,1 mg/mL) coverslips and synchronized as described. 17,5 hours after the thymidine block release coverslips were transferred to growth medium supplemented with 55 mM NaCl or 55 mM KCl. The cells were then incubated for 10 minutes at 37°C and 10% CO_2_ and subsequently placed on ice. The growth medium was immediately replaced by ice-cold PBS supplemented with 0,5 mM MgCl_2_ and 1 mM CaCl_2_ (PBS-Mg-Ca). After 5 minutes of incubation, the PBS-Mg-Ca was replaced by fresh PBS-Mg-Ca supplemented with 0.2% gelatine and anti-SgII antibody 718 [20]. After 30 minutes of incubation at 0°C, cells were washed twice with ice-cold PBS-Mg-Ca/gelatine 0.2% and fixed with ice-cold 4% PFA/4% sucrose for 15 minutes at room temperature. From here all further steps were carried out at room temperature. The fixative was quenched with 50 mM NH_4_Cl, cells were washed once in PBS-Mg-Ca/gelatine 0.2%, then incubated for 20 minutes in PBS-Mg-Ca/gelatine 0.2% supplemented with the secondary antibody (goat-anti-rabbit-TRITC) and Hoechst dye, followed by mounting in Moviol.

### 4.7. Quantification of Surface-Exposed SgII Signals

To compare the rate of exocytosis between interphase and metaphase cells, we applied a semi-automated application written in MATLAB. Synchronized monolayers of PC12 cells were immunolabeled for SgII and with Hoechst as described above. In parallel a wheat germ agglutinin (WGA) surface staining was performed (WGA-Alexa Fluor 488, 500 ng/mL). Metaphase cells, identified by the Hoechst stain, were chosen at random and the three channels for SgII (excitation 555 nm), WGA (excitation 488 nm) and Hoechst (excitation 400 nm), respectively, were recorded at the cell midzone using a widefield imaging setup. Interphase cells visible in the same optical plane were used as internal control for each metaphase cell (see below). As a first step in the quantification protocol the uniform WGA surface staining was used for automated cell segmentation [27], (Figure 10(A), 10(C)). The identified cell borders at the outside of the cell clusters were extended by 3 pixels to each side and the resulting mask was superimposed with the SgII channel (Figure 10(F)). The total intensity of the SgII signal inside the mask divided by the pixel area of the mask was calculated. Due to the strong variability of the SgII signals at the cell borders located inside the cell clusters, they were not considered for the quantification. From the obtained average pixel value for SgII under stimulated conditions the similarly identified average pixel value of unstimulated cells was subtracted as a background.

Average pixel values ≤0 after background subtraction were classified as cells that did not respond to the stimulus. The ratio of cells with intensity values ≥0 divided by the total number of cells revealed the percentage of cells that responded to the stimulus.

## Supplementary Material

Movie SM1. A PC12 cell coexpressing hCgB-DsRedExpress (red) and EGFP-tubulin (green) is observed as it progresses through cell division. Indicated time refers to “hrs:min:sec, msec”.
Movie SM2. A PC12 cell coexpressing hCgB-DsRedExpress (red) and EGFP-tubulin (green) is observed as it progresses through late stages of mitosis. Single time frames from this movie were used to generate Figure 7. Indicated time refers to “hrs:min:sec, msec”.
Movie SM3. A PC12 cell expressing hCgB-DsRedExpress (grayscale) is recorded as it progresses through cytokinesis. Indicated time refers to “hrs:min:sec, msec”.
